# An MILP-Based Cross-Layer Optimization for a Multi-Reader Arbitration in the UHF RFID System

**DOI:** 10.3390/s110302347

**Published:** 2011-02-24

**Authors:** Jinchul Choi, Chaewoo Lee

**Affiliations:** Graduate School of Information and Communication, Ajou University, Suwon 443-749, Korea; E-Mail: rockerjc@ajou.ac.kr

**Keywords:** RFID, anti-collision, multi-reader arbitration, cross-layer optimization, MILP

## Abstract

In RFID systems, the performance of each reader such as interrogation range and tag recognition rate may suffer from interferences from other readers. Since the reader interference can be mitigated by output signal power control, spectral and/or temporal separation among readers, the system performance depends on how to adapt the various reader arbitration metrics such as time, frequency, and output power to the system environment. However, complexity and difficulty of the optimization problem increase with respect to the variety of the arbitration metrics. Thus, most proposals in previous study have been suggested to primarily prevent the reader collision with consideration of one or two arbitration metrics. In this paper, we propose a novel cross-layer optimization design based on the concept of combining time division, frequency division, and power control not only to solve the reader interference problem, but also to achieve the multiple objectives such as minimum interrogation delay, maximum reader utilization, and energy efficiency. Based on the priority of the multiple objectives, our cross-layer design optimizes the system sequentially by means of the mixed-integer linear programming. In spite of the multi-stage optimization, the optimization design is formulated as a concise single mathematical form by properly assigning a weight to each objective. Numerical results demonstrate the effectiveness of the proposed optimization design.

## 1. Introduction

Radio frequency identification (RFID) is a non-contact technology that helps machines or computers identify objects, record metadata or control individual target through radio wave. Essentially, RFID is a technology that connects objects to Internet, so the objects can be tracked and their information can be shared. The concept of RFID technology is simple: Place a tag, *i.e.*, a microchip with an antenna, on an item and then use a reader device to read data off of the tag through RF links. The reader passes the information to operators so that the data can be used to create business value. Since there are many different types of RFID systems according to frequency bands and types of tags, it is important to choose the right type of RFID system for a particular application. The basics of RFID technology and current standards can be found in [1].

Recently, ultra high frequency (UHF) band passive RFID systems, which operate in the 860–960 MHz, have received considerable attention. It is generally accepted that the UHF RFID system connected to intelligent wireless sensor network can revolutionize commercial processes or present many opportunities for process improvement such as supply-chain management [2]. Indeed, a number of retail organizations have adopted or planned to adopt the UHF RFID system in their supply chains. In these deployments, a number of readers may be in operation at the same time and the readers with overlapping interrogation zones can interfere with each other. This will often reach a point where readers are unable to recognize any tag located within their respective interrogation zones. The readers may also interfere with other’s operation even though their interrogation zones do not overlap [3].

There are two primary types of controllable reader interference in the UHF RFID system; reader-to-tag interference and reader-to-reader interference [4]. Reader-to-tag interference occurs when a tag is located in the interrogation zones of two or more readers and more than one reader attempts to interrogate the tag at the same time. This type of interference causes the tag to behave and communicate in undesirable ways. On the other hand, the reader-to-reader interference occurs when a reader transmits a signal that interferes with the operation of another reader; thus preventing the other reader from communicating with tags in its interrogation zone.

The reader interference problem can be solved by output signal power control of each reader, spectral and/or temporal separation among the interfering readers. The system performance in terms of tag recognition rate, interrogation coverage and delay, reader utilization, *etc.*, depends on how to adapt the reader arbitration metrics such as time, frequency, and output power to the system environment. However, the optimization problem with consideration of the diverse arbitration metrics is complicated and difficult to be extracted and solved. Thus, most of the proposed strategies [3–26] have solved the reader interference problem with consideration of one or two arbitration metrics. For example, frequency-hopping spread spectrum (FHSS) utilizes multiple frequency channels, while DCS, VDCS (Colorwave) [5,6] and listen before talk (LBT) [7] require readers to operate at different times. DAPC and PPC [8,9] dynamically adjust transmission power at discrete-time steps using the SNR of backscatter signal measured at each reader. Although the existing proposals may be effective in preventing the reader interference problem, they may not be the best solution due to insufficient arbitration metrics.

In this paper, we introduce a novel cross-layer optimization design based on mixed-integer linear programming (MILP), which optimally assigns communication channel, time, and output power to each RFID reader. The solution from the optimization design not only solves the reader interference problem, but also achieves multiple objectives such as minimum interrogation cycle, maximum reader utilization, and energy efficiency. Based on the priority of the multiple objectives, our cross-layer design is presented as a three-stage optimization problem. In the first stage, we optimize the RFID system to have minimum cycle time. Here, each RFID reader should be scheduled to successfully recognize the tags within its desired interrogation range at least once during a cycle. Once the temporal schedule is assigned, the RFID readers repeat the same temporal pattern after one cycle. Our first aim is to minimize this cycle so that interrogation delay of RFID reader can be minimal. In general, the solution, *i.e.*, resource assignment set, to the first stage that provides minimum cycle time is not unique. Thus, among the multiple solutions, it is desirable to select a set that provides the maximum utilization. In the second stage, we optimize the system to achieve the maximum utilization while maintaining the minimum cycle time found in the first stage. Again the system can be optimized in terms of output power while maintaining the optimality found in the previous stages. Then, we transform the three-stage optimization problem into a compact single-stage optimization problem by properly assigning a weight to each of the three objectives, because the three-stage optimization is cumbersome to execute.

The rest of the paper is organized as follows. The existing proposals to solve the RFID reader interference problem are reviewed in Section 2, and the network model and interference model are introduced in Section 3. The cross-layer optimization designs are presented in Section 4. Numerical results and comparative view of the anti-collision approaches are shown in Sections 5 and 6. Finally, we give our concluding remarks in Section 7.

## 2. Countermeasures To Reader Interference Problem

**Figure 1.**
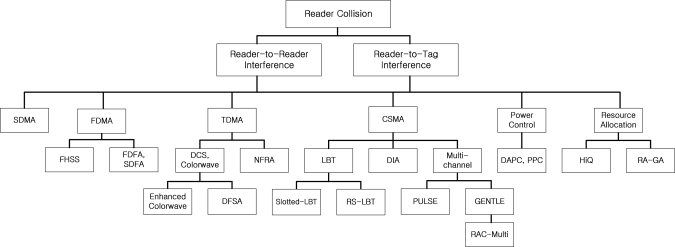
Taxonomy of anti-collision solutions for RFID readers.

Interference caused by the operation of an RFID reader is referred to as a reader collision [4]. Reader collisions prevent the colliding readers from communicating with the RFID tags in their respective interrogation zones. To deal with the reader collision problem, there have been various works and these works, which can be categorized as either SDMA, FDMA, TDMA, CSMA, power control, and resource allocation. The classification and solutions of the reader collision problem are shown in Figure 1. In the following subsections, we review the solutions.

### 2.1. SDMA based collision resolution

Space division multiple accesses (SDMA) relates to techniques that reuse channel capacity in spatially separated areas. The simplest solution is to significantly reduce the range of a single reader, but it requires a number of readers to cover the operation field. Another solution is to use an electronically controlled directional antenna on the reader, the directional beam can be pointed directly at a tag. Thus, various tags can be differentiated by their angular position in the interrogation zone of the reader. However, the SDMA technique requires the relatively high implementation cost of the complicated antenna system.

### 2.2. FDMA based collision resolution

RFID standards such as ISO/IEC 18000-6 [10] and EPC Class 1 Gen. 2 [11] basically use spectral planning. This method spectrally separates the reader interrogation and the tag reply, which may cause the tag collision and the reader collision. To prevent such collisions, FHSS that randomly switches carriers among frequency channels is utilized in the standards. However, since the tags do not have any frequency selectivity, multiple reader-to-tag interference problems still exist in these standards. Furthermore, the readers may suffer from persistent reader collision when a number of RFID readers operate in a region.

Fully distributed frequency allocation (FDFA) and semi distributed frequency allocation (SDFA) [12] are optimization-based distributed channel selection and randomized interrogation algorithms for dense RFID systems. For a proper channel selection, the multi-channel randomized interrogation problem is formulated as an optimization problem. The objective of the problem is to achieve max-min fair resource allocation among the readers by taking into account reader-to-reader and reader-to-tag interference. Since the problem is not a convex optimization problem, finding the optimal solution is not easy in general. Thus, distributed algorithms have been developed to reach the solution.

### 2.3. TDMA based collision resolution

In TDMA based collision resolution schemes, transmission time is divided into frames with several fixed-length time slots. To avoid simultaneous transmissions each reader is required to operate at a different time slot in a frame. To execute the slot distribution a pair of distributed algorithms called DCS and VDCS (or Colorwave) [5,6] have been introduced. In these algorithms, each reader randomly chooses a time slot and communicates with tags only at its slot. If the reader collision occurs, the reader selects a new time slot and sends a kick message to all its neighbors to indicate its new slot. The switch and reservation action is referred as the kick message. If any neighbor has the same slot, it chooses a new slot and sends a kick. This repeats until all the tags are recognized.

In DCS, the frame size is fixed and thus its implementation is simple. However, the performance is decreased when the frame size does not match the number of readers. For example, if the frame size is small and the number of active readers is large, then the efficiency may be low due to heavy collisions. The opposite may be also inefficient due to lots of idle slots. To solve this problem, Colorwave allows each reader to dynamically change its frame size. In Colorwave, each reader monitors the percentage of successful transmissions during a particular time period. If the percentage of successful transmission goes below a lower threshold, the frame size is incremented. If the percentage increases beyond an upper threshold, the frame size is decremented. Since Colorwave builds upon DCS, the process to solve the reader collision is the same as that of DCS.

Colorwave is an effective algorithm to avoid collisions based upon local information. However, in Colorwave the readers may experience a number of collisions until it eventually reaches the steady state. The major drawback of the algorithm is that it takes some time for readers to find appropriate frame size. Furthermore, each reader cannot determine whether current frame size is optimal and it may keep changing its frame size. To cope with the oscillation problem, Enhanced colorwave was proposed [13]. The Enhanced Colorwave algorithm requires the readers to synchronize the frame size through the kick messages and to exponentially increase the time interval in changing the frame size when the frame size oscillates. As a result, the frame size of all the readers converges to an optimum and the oscillation in frame size decreases as time passes. When Colorwave applies to mobile RFID readers, since the readers keep moving, reader collisions can occur frequently and so the frame size can become unnecessarily high. Therefore, Colorwave and Enhanced colorwave are appropriate in situations where the RFID readers are fixed or barely move. For mobile RFID readers, the dynamic frame size adjustment (DFSA) [14] algorithm automatically adjusts the frame size of each reader without using manual parameters by adopting the dynamic frame size adjustment strategy when collisions occur at a reader.

Neighbor friendly reader anti-collision (NFRA) [15] is a reader anti-collision algorithm using a polling server in dense RFID networks with mobile readers. In NFRA, transmission time is partitioned into fixed-length rounds. In the beginning of a round, a polling server broadcasts a random number and then the readers compare their random numbers with the random number from the server. If they are the same, the readers decide to actively operate during a current round and issue beacons. Since multiple readers decide to operate and issue beacons at the same time, the readers monitor whether their beacons collide with others. After confirming the non-occurrence of collision, the readers read tags during a current round.

### 2.4. CSMA based collision resolution

In the European regulation as outlined in ETSI EN 302 208 [7], a reader must listen on the data channel for a specified minimum time to confirm that the data channel is idle before the reader uses the channel. If the channel is idle, the reader starts interrogating tags through the channel. If the channel is not idle, the reader chooses a random back-off and then listens again. This is called listen before talk (LBT). When LBT is applied to the multi-channel system, not all the channels can be fully utilized because a number of readers may compete on a particular channel [16].

Slotted-LBT [17] is a TDMA-based LBT scheme to reduce the time variance of the channel access and interference effect. In Slotted-LBT, each time frame consists of several fixed-size time slots and the readers acquire data channels using the LBT scheme. Since the conventional LBT scheme does not provide any fairness mechanism for the readers, some readers may spend much more time to acquire data channels in dense reader RFID networks. To reduce the time variance of the channel access, Slotted-LBT has an algorithm to disperse the readers to the slot. It also reduces the frequency interference among adjacent readers by the means of spatial zonation. However, maintaining synchronization requires extra management overhead.

Reader synchronized(RS)-LBT schemes [18] are techniques that coordinate the operation of multiple readers so that they share the same channels while observing the rules for the LBT. For example, all readers in the system are connected to a predefined network controller. Then, the network controller scans all the data channel and assigns available channel and operation time to each reader. When the network controller is absent, each reader becomes a master reader in turn and manages the synchronization process.

PULSE [19] and GENTLE [20] are reader collision avoidance schemes based on periodic beaconing on a separate control channel. PULSE is developed based on the assumption that there are only two channels; a control channel and a data channel. When a reader communicates with tags, it periodically broadcasts a beacon message through the control channel. The readers receiving the beacon message are prohibited from tag interrogation until they no longer receive beacon messages. In contrast, GENTLE considers multiple data channels because the international standards often do not restrict the number of data channels [10,11,18,21]. In GENTLE, the way that a beacon message is sent to other neighbor readers is basically the same with PULSE, but the beacon message is restricted to be sent only within a certain distance. As a result, readers avoid multiple reader-to-tag collision using beacon messages when they are close one another, and reader-to-reader collision using multiple data channels when the distance between those readers is long. Besides, to extend the reader coverage, each reader can embed tag information in its beacon message and share it among neighbor readers. Since each reader randomly selects a channel among available data channels, this may lead to the adjacent channel interference between neighboring readers. To cope with the problem, RAC-Multi [22] separates data channels into odd- and even-numbered channels and uses the odd-numbered channels first instead of randomly selecting a channel from all available channels. RAC-Multi also provides one channel of separation between the control channel and data channels to ensure that interference between control messages and the signal of the adjacent channel does not occur.

Tanaka and Sasase [23] proposed two distributed interference avoidance (DIA) algorithms based on the detect-and-abort principle for multi-channel readers. They formulated an LP-based RFID system model and derived the optimum communication probability of the readers for a given reader deployment scenario. Based on the derived communication probability of the readers and the interference detected for a predetermined period, the first algorithm determines how each reader should communicate with tags. For the interference detection, a contention-based reader-to-tag interference detection scheme is used. The second algorithm effectively avoids the asymmetric interferences by adding a simple centralized control of each reader’s transmit duty.

### 2.5. Power control based collision resolution

Recent works have explained that higher interference merely causes a reduction in the interrogation range of the RFID reader [3,8,9,24]. In a passive RFID system, to successfully recognize tags, the signal to interference plus noise ratio (SINR) of the signal backscattered from a tag must meet a required threshold, which depends on desired read rate and BER. The SINR measured at a reader is influenced by various factors such as the reader signal power, interference power, and distance between the reader and a target tag, and others. Thus, a desired interrogation range and read rate may be achieved with a proper signal power control.

In [8,9], two distributed power control schemes have been proposed; DAPC and PPC. In these schemes, each reader dynamically adjusts its output power at discrete-time steps using the SINR of backscatter signal measured at each reader. The DAPC algorithm consists of two building blocks; adaptive power update and selective back-off. The goal of the adaptive power update is to achieve the required SINR with an appropriate output power by correctly estimating the interference power of next time step. In dense networks, DAPC tends to make each reader to constantly increase its power. Thus, not all readers can simultaneously achieve the target SINR. To cope with the problem, a selective random back-off policy forces high power reader to stop transmission for a certain back-off time so that other readers can achieve required SINR.

On the other hand, PPC attempts to temporally separate the readers by appropriately assigning a time period to each of them, along with the power control. However, any scheme to adaptively distribute the time period is not provided due to the complexity of the problem. Consequently, PPC is only implemented using a fixed power distribution and distribution sets obtained from a neural network, which is one of heuristic methods.

### 2.6. Centralized resource allocation based collision resolution

When a number of RFID readers operate in a region, the performance of distributed anti-collision schemes may reduce [20,25]. In this case, a centralized reader anti-collision scheme may be more effective. The centralized scheme analyzes the various factors affecting the collision and solves the problem using resource allocation in which proper frequencies and time are allocated to the readers.

HiQ [26] is an hierarchical Q-learning algorithm. To find the optimal solution, it uses Q-server as the coordinator to conduct the Q-learning algorithm [27], which is a form of reinforcement learning, with the collision information of readers. The HiQ allocates channels and operation time to the readers to maximize the number of operating readers at the same time. To allocate proper resources to the readers, the HiQ utilizes the collision information of neighboring readers. Q-learning assumes collision detection for readers which are not in sensing range of each other. However, each reader may not detect all collisions and incorrect operation of the scheme may occur. Furthermore, when the number of readers is large, it may be difficult to find the optimal solution due to large complexity.

RA-GA [25] is a resource allocation technique based on a heuristic method. Achieving the SINR constraint of each reader, the RA-GA appropriately assigns spectral and temporal resources, *i.e.*, channel and time, to each reader to maximize the total area covered by readers during a certain time period by means of the genetic algorithm. However, it takes time to find the best solution and the solution may not be the global optimum due to the nature of the heuristic method.

Most solutions to the reader collision problem do not simultaneously consider various reader arbitration metrics such as channel, time, and power. For more effective anti-collision solution, it is desirable to analytically model the reader interference with consideration of the various metrics. Then, based on the analytic models, useful optimization schemes can be extracted and solved. In the next section, the reader-to-reader interference model to analyze the relationship between the interference powers from multiple readers and the interrogation range of a desired reader, and the network model for the problem formulation are introduced.

## 3. Network Model

### 3.1. General description

Consider a passive RFID system in which *R* readers share *C* communication channels. Let Ω*_R_* and Ω*_C_* the sets of readers and available communication channels, respectively. In this system, both FDMA and TDMA schemes can be used to reduce the interference among readers more effectively. In a TDMA schedule, time is partitioned into fixed size of frames. Each frame consists of *S* time slots and let Ω*_S_* denote the set of time slots. The goal of our optimization design is to derive an optimal resource allocation solution, which enables the readers to work without interfering one another and to achieve the multiple objectives. We assume that the solution is to operate the readers during a frame and the same resource allocation pattern repeats from frame to frame so that we focus on the resource scheduling done in one frame only.

Let $\gamma_{i}^{c,s}$ and $P_{i}^{c,s}$ respectively indicate the state and the output power of reader *i* ∈ Ω*_R_* = {1, 2, ...,*R*} communicating on channel *c* ∈ Ω*_C_* = {1, 2, ...,*C*} at slot *s* ∈ Ω*_S_* = {1, 2, ...,*S*}. Let *P*_min_ and *P*_max_ denote the minimum output power required for the tag operation and the limit on the maximum output power, respectively. For example, $\gamma_{k}^{m,n}=1$ indicate that reader *k* uses channel *m* is active at slot *n* and the corresponding output power is $P_{k}^{m,n}\in \left[P_{\text{min}},\;P_{\text{max}}\right]$; otherwise, $\gamma_{k}^{m,n}=0$ and $P_{k}^{m,n}=0$. Here, we assume the readers are powered by unlimited sources and consequently we do not consider the lifetime of the RFID readers.

As the requirement of active readers, the reply of any tag within their interrogation ranges should be successfully decodable. That means that the SINR of a signal backscattered from the tag should meet a given threshold. The SINR of the backscattered signal is influenced by the output power of the reader, interference from the other readers and others. In the next sub-section, we derive the reader interference model to determine active readers and their corresponding powers.

### 3.2. Reader interference model

In a passive RFID system, to successfully recognize a tag, the SINR of a signal backscattered from the tag must exceed a threshold, which depends on the tag encoding method and the desired BER. Let *SINR_A_* denote the SINR measured at reader *A*. To successfully recognize a tag, the following must be satisfied.

(1) $${\mathrm{SINR}}_{A}=\frac{S_{A}}{I_{\mathrm{BA}}+N_{0}}\geq \Gamma$$

where *S_A_* and *I_BA_* respectively denote the power backscattered from the tag and the interference power from reader *B* measured at reader *A*. *N*_0_ denotes the background noise power and Γ represents the required SINR threshold. Since the tag reflects a fraction of the power received from reader *A*, *S_A_* can be modeled in terms of the output power of reader *A*, the distance *x_A_* between reader *A* and the tag, and other factors [3]. It is primarily based on the Friis transmission equation and given by

(2) $$S_{A}\;\left(x_{A}\right)=\alpha_{\text{BW}}E_{\text{tag}}P_{A}G_{T}G_{R}{\left(\frac{\lambda_{A}}{4\pi x_{A}}\right)}^{4}$$

where *P_A_* denotes the output power of reader *A*, *E*_tag_ is the effective power reflection coefficient of a tag, *λ_A_* is the wavelength used by reader A, and *G*_T_ and *G*_R_ are the gains of the transmit antenna and the receive antenna, respectively. As the received signal travels to and from the tag, it experiences channel path-losses. Since the forward and backward path-losses are identical, the total path-loss becomes ${\left(\frac{\lambda_{A}}{4\pi x_{A}}\right)}^{4}$. As the path between the reader and the tag is short and is a line-of-sight (LOS) path, fading effects can be ignored [24]. Additionally, *α*_BW_ denotes the fractional power ratio in the bandwidth that is used. *α*_BW_ can be expressed, using the power spectral density (PSD) function Φ(*f*) of the signal from the tag, as $\alpha_{\text{BW}}=\int_{\text{BW}}\Phi \left(f\right)\mathrm{df}/\int_{-\infty }^{\infty }\Phi \left(f\right)\mathrm{df}$; where BW denotes the channel bandwidth. The PSD of the signal is related to its data-encoding scheme, and FM0 and Miller subcarrier sequence codes are adopted for the tag encoding scheme in EPC Class 1 Gen. 2 specification [11]. The normalized spectrum power *α*_BW_ is approximated to 0.86 for FM0 code and 0.78 for Miller subcarrier sequence code, respectively [24].

For the tag operation, each tag must be supplied with the energy more than that of the threshold power, which is determined according to the chip design of the tag and matching condition of the antenna. The minimum output signal power of reader *A* to recognize a tag located at distance *x_A_* from reader *A*, $P_{\text{min}}^{A}\left(x_{A}\right)$, is given by

(3) $$P_{\text{min}}^{A}\left(x_{A}\right)=\frac{P_{\text{TH}}}{\alpha_{\text{BW}}G_{T}}{\left(\frac{4\pi x_{A}}{\lambda_{A}}\right)}^{2}$$

where *P*_TH_ denotes the threshold power, *i.e.*, the minimum power required for the tag operation. The reported values range from −20 to −15 dBm [28,29].

The interference power from reader *B* measured at reader *A*, *I_BA_*, can be also modeled in terms of the output power of reader *B*, the distance *d_BA_* between readers *B* and *A*, and other factors as follows [3].

(4) $$I_{\mathrm{BA}}\left(d_{\mathrm{BA}}\right)=h\beta_{\text{mask}}^{\mathrm{BA}}P_{B}G_{T}G_{R}{\left(\frac{\lambda_{B}}{4\pi d_{\mathrm{BA}}}\right)}^{2}$$

where *h* and $\beta_{\text{mask}}^{\mathrm{BA}}$ denote the fading coefficient and the spectrum mask level as a function of the frequency separation between desired reader *A* and interfering reader *B*, respectively. In telecommunications, out-of-band radio-frequency emissions of transceivers are common and RFID readers are no exception. Since the signals backscattered from tags are weak, they can be interfered by the minor out-of-band radio-frequency emissions from other readers. To deal with such adjacent channel interference, the out-of-band radio-frequency emissions of RFID readers are constrained by the spectrum mask requirements, which is the power contained in a specified frequency bandwidth at certain offsets relative to the total carrier power [11,18,21]. Let *f_A_* and *f_B_* denote the center frequency of the channel assigned to readers *A* and *B*, respectively. Then, the spectrum mask level $\beta_{\text{mask}}^{\mathrm{BA}}$ is given by $\beta \left(\frac{\left|f_{A}-f_{B}\right|}{\mathrm{BW}}\right)$. According to the CEPT dense-reader environment [11], when interfering readers use adjacent channels, *i.e.*, *β*(1), the spectrum mask for the readers is 1/1000. In the case that the readers are distant from each other, the interference between the readers may be very small. However, if the readers are close to each other, the interference cannot be ignored. Therefore, to more effectively mitigate the interference among neighboring readers operating at the same, it is desirable to allocate the channels sufficiently separated from each other to the readers.

So far, we have presented an FDMA-based interference model with two readers. In case of the FDMA-based anti-collision scheme, when the number of active readers is larger than that of available channels, the readers may suffer from persistent reader collision. Thus, we need to employ the TDMA scheme also to eliminate more readers from competing with each other. In adopting TDMA scheme, we have the SINR constraint for an active reader *i* with the interrogation range *x_i_* as follows.

(5) $$\frac{\kappa_{1}P_{i}^{c,s}/x_{i}^{4}}{\kappa_{2}\sum_{j\neq i}P_{j}^{c,s}\beta_{\text{mask}}^{\mathrm{ji}}/d_{\mathrm{ji}}^{2}+N_{0}}\geq \Gamma ,\;\;i,\;j\;\in \;\Omega_{R},\;c\;\in \;\Omega_{C},\;s\;\in \;\Omega_{S}$$

where constants *κ*_1_ and *κ*_2_ represent $\frac{\alpha_{\text{BW}}E_{\text{tag}}G_{T}G_{R}\lambda_{i}^{4}}{{\left(4\pi \right)}^{4}}$ and $\frac{hG_{T}G_{R}\lambda_{j}^{2}}{{\left(4\pi \right)}^{2}}$, respectively.

## 4. A Cross-layer Optimization for Resource Scheduling and Power Allocation

In this section, we propose a novel MILP based cross-layer optimization design, which optimally assigns communication channel, time, and output power to each RFID reader. Based on the priority of the multiple objectives, our cross-layer design is presented as a three-stage problem, which sequentially optimizes the system. However, this approach is cumbersome to reach the optimal solution due to the nature of multi-stage problem. Thus, we propose an equivalent single-stage problem and prove that the three-stage problem can be converted to a compact single-stage problem by properly assigning a weight to each of the three objectives.

### 4.1. Three-stage optimization

Since each reader must be active at least in a frame and the communication schedules repeat the same temporal pattern after one frame, the maximum waiting time for the next service is determined by the frame size. We put the highest priority to minimize the frame size in the cross-layer optimization.

After the minimum frame size is found, our next priority is to find the maximum reader utilization while maintaining the minimum frame size obtained in the first stage. The utilization is defined as the total number of active time slots in a frame. Finally, our last goal is to control the output power so that the minimum power can be assigned to the readers while maintaining the frame size and the utilization obtained from previous stages.

#### (1) Minimization of interrogation cycle

In the first stage, the objective is to find the minimum frame size, S^*^, during which each reader must be activated at least once. The first stage optimization problem is formulated as follows.

(6a) $$\underset{\left\{P_{i}^{c,\;s}\right\},\;\left\{\gamma_{i}^{c\;,s}\right\},S}{\text{Min}}S$$

subject to:

(6b) $$\sum_{k=1}^{S}\sum_{m=1}^{C}\gamma_{i}^{m,\;k}\geq 1,\;\;\;i\;\in \;\Omega_{R}$$

(6c) $$\sum_{k=1}^{C}\gamma_{i}^{k,\;s}\leq 1,\;\;\;i\in \Omega_{R},\;s\;\in \;\Omega_{S}$$

(6d) $$\kappa_{1}\frac{P_{i}^{c,s}}{x_{i}^{4}}\geq \Gamma \cdot \left(\kappa_{2}\sum_{j\neq i}\frac{\beta_{\text{mask}}^{\mathrm{ji}}P_{j}^{c,s}}{d_{\mathrm{ji}}^{2}}+N_{0}\right)+D\left(\gamma_{i}^{c,s}-1\right),\;i,\;j\;\in \;\Omega_{R},\;c\;\in \;\Omega_{C},\;s\;\in \;\Omega_{S}$$

(6e) $$\gamma_{i}^{c,s}P_{\text{min}}^{i}\left(x_{i}\right)\leq P_{i}^{c,s}\leq \gamma_{i}^{c,s}P_{\text{max}},\;\;\;i\;\in \;\Omega_{R},\;c\;\in \;\Omega_{C},\;s\;\in \;\Omega_{S}$$

(6f) $$\gamma_{i}^{c,s}\in \left\{0,1\right\},\;\;\;\;\;i\;\in \;\Omega_{R},\;c\;\in \;\Omega_{C},\;s\;\in \;\Omega_{S}.$$

Constraint (6b) requires each reader to be activated at least once in a frame, and constraint (6c) requires each reader only to use a single channel when it is active. Constraint (6d) is equivalent to (5), in which *D* is a constant that satisfies

(7) $$D\geq \Gamma \left(\kappa_{2}\underset{j,i}{\text{max}}\sum_{j\neq i}\frac{P_{\text{max}}}{d_{\mathrm{ji}}^{2}}+N_{0}\right).$$

Constraint (6d) is equivalent to (5) for the following reasons.

- When reader *i* is scheduled to communicate in channel *c* at time slot *s*, *i.e.*, $\gamma_{i}^{c,s}=1$, constraint (6d) can be exactly be rewritten as (5).
- When reader *i* is not scheduled, *i.e.*, $\gamma_{i}^{c,s}=0$, constraint (6d) is satisfied if (7) holds.

Therefore, constraints (5) and (6d) are equivalent. The problem (6a)–(6f) is a mixed-integer linear programming (MILP) problem. Software packages such as LINGO [30] and CPLEX [31] are available to solve the proposed MILP problem. However, the problem (6a)–(6f) may have more than one feasible solutions that offer the same minimum frame size. Thus, among the various solutions, it is desirable to find solutions which maximize the utilization of RFID readers. This will be done in the second stage of the optimization.

#### (2) Maximization of RFID reader utilization

We define the utilization of the RFID readers as the total number of active time slots for all the readers. Let U^*^ be the maximum utilization found in the second stage. Note that our second goal is to maximize the utilization while maintaining the minimum frame size obtained in the first stage. Then, the second stage optimization problem is formulated as follows.

(8a) $$\underset{\left\{P_{i}^{c,s}\right\},\left\{\gamma_{i}^{c,s}\right\},U}{\text{max}}U$$

subject to:

(8b) $$U=\sum_{k=1}^{R}\sum_{m=1}^{C}\sum_{n=1}^{S}\gamma_{k}^{m,n}$$

(8c) $$S=S^{*}$$

(8d) Constraints (6b)–(6f).

Substituting *W* = −*U* the optimization problem (8a)–(8d) can be rewritten as

(9a) $$\underset{\left\{P_{i}^{c,s}\right\},\left\{\gamma_{i}^{c,s}\right\},W}{\text{min}}W$$

subject to:

(9b) W=−U

(9c) Constraints (8b)–(8d)

which is an MILP problem as well. The solution to the problem (9a)–(9c) may not be unique either because it is still available to control the output signal power of each RFID reader. As the last step in the cross-layer optimization, we optimize the output power of the RFID readers.

#### (3) Minimization of RFID reader power consumption

The objective in the last stage is to minimize the total output power of the RFID readers while maintaining the optimality found in the first and second stages. The last stage optimization problem is formulated as follows.

(10a) $$\underset{\left\{P_{i}^{c,s}\right\},\left\{\gamma_{i}^{c,s}\right\},E}{\text{min}}E$$

subject to:

(10b) $$E=\sum_{k=1}^{R}\sum_{m=1}^{C}\sum_{n=1}^{S}P_{k}^{m,n}$$

(10c) $$\sum_{k=1}^{R}\sum_{m=1}^{C}\sum_{n=1}^{S}\gamma_{k}^{m,n}=U^{*}$$

(10d) Constraints (8c), (8d).

Among all the scheduling and power allocation sets, *i.e.*, $\left\{\gamma_{i}^{c,s}\right\}$ and $\left\{P_{i}^{c,s}\right\}$, we obtain the optimal set whose the associated subproblem (10a)–(10d) has the minimal value.

### 4.2. Single-stage optimization

The three-stage design sequentially optimizes the RFID system based on the priority of the multiple objectives. This is a bit inconvenient to derive a final solution. For simplicity of the derivation, we show the following key result.

**Theorem 1.** *The three-stage optimization problem (6a)–(6f), (9a)–(9c), and (10a)–(10d) is equivalent to the following single-stage optimization problem:*

(11a) $$\underset{\left\{P_{i}^{c,s}\right\},\left\{\gamma_{i}^{c,s}\right\},S,W,E}{\text{min}}S+\xi_{1}(W+\xi_{2}E)$$

subject to:

(11b) Constraints (6b)–(6f), (8b), (9b), (10b)

where $\xi_{1}\in \left(0,\frac{1}{R^{2}-R+1}\right]$ and $\xi_{2}\in \left(0,\frac{1}{R\;\left(P_{\text{max}}\;R-P_{\text{min}}\right)}\right)$ are constant.

*Proof.* We show that the solution to the single-stage problem maintains the identical optimality obtained in each stage of the three-stage problem. Thus, the proof consists of three steps.

In the first step, we prove that the solution of single-stage problem (11a), (11b) and the solution of problem (6a)–(6f), the first stage of the three-stage problem, will give the same minimum frame size. Let (*S*^+^, **P**^+^, **Γ**^+^) be a solution to problem (6a)–(6f), with *S*^+^ being the frame size, **P**^+^ and **Γ**^+^ being the corresponding power allocation matrix and the reader schedule matrix, respectively. Note that there may be many optimal solutions to problem (6a)–(6f) with the same optimal value *S*^+^. Therefore,(*S*^+^, **P**^+^, **Γ**^+^) may be one of them.

Similarly, let (S^*^, P^*^, Γ^*^, W^*^, E^*^) be an optimal solution to the single-stage problem, with S^*^ being the frame size, P^*^ and Γ^*^ being the corresponding power allocation matrix and reader schedule matrix, respectively. Furthermore, let W^*^ and E^*^ be the negative utilization and the normalized energy consumption of all the readers, respectively. The optimal objective value of the single-stage (11a)–(11b) is given by

(12) $$v^{*}=S^{*}+\xi_{1}\left(W^{*}+\xi_{2}E^{*}\right)$$

In the following, we prove that S^*^ = *S*^+^ using contradiction. Suppose S^*^ > *S*^+^. Since both S^*^ and *S*^+^ are integers, we have

$$S^{*}-S^{+}\geq 1$$

Define

$$\begin{array}{l} W^{+}=-\sum_{k=1}^{R}\sum_{m=1}^{C}\sum_{n=1}^{S^{+}}{\left(\gamma_{k}^{m,n}\right)}^{+}\\ E^{+}=\sum_{k=1}^{R}\sum_{m=1}^{C}\sum_{n=1}^{S^{+}}{\left(P_{k}^{m,n}\right)}^{+} \end{array}$$

Then, we have

(13) $$-R^{2}\leq W^{+}\leq -R$$

(14) $$P_{\text{min}}R\leq E^{+}\leq P_{\text{max}}\;R^{2}$$

Equation (13) holds for the following reasons.

- The minimum utilization is identical with the number of readers because each reader should be scheduled at least once in a frame. Thus, the utilization is not less than the number of readers.
- When each reader is spatially separated enough, all the readers may be scheduled at a time slot regardless of the number of available channels. Since the maximum frame size is set to the number of readers, the utilization does not exceed the square of the number of readers.

Equation (14) holds because the total energy consumption can be expressed by the product of the average energy consumed by each reader and the utilization. Similarly, the boundaries of W^*^ and E^*^ are equivalent with those of *W*^+^ and *E*^+^.

Apparently, (*S*^+^, **P**^+^, **Γ**^+^, *W*^+^, *E*^+^) satisfies constraints (6b)–(6f), and thus, it is a feasible solution to the single-stage problem with the objective value being

(15) $$v^{+}=S^{+}+\xi_{1}\left(W^{+}+\xi_{2}E^{+}\right)$$

Furthermore, we have

(16) $$\begin{array}{ll} v^{*}-v^{+} & =S^{*}-S^{+}-\xi_{1}\left\{\left(W^{+}-W^{*}\right)+\xi_{2}\left(E^{+}-E^{*}\right)\right\}\\ & \geq 1-\xi_{1}\left\{\left(R^{2}-R\right)+\xi_{2}\left(P_{\text{max}}\;R^{2}-P_{\text{min}}R\right)\right\}\\ & >1-\xi_{1}\left\{R^{2}-R+1\right\}\\ & \geq 0 \end{array}$$

The first inequality in (16) holds because

$$\begin{array}{l} S^{*}-S^{+}\geq 1\\ W^{+}-W^{*}\leq R^{2}-R\\ E^{+}-E^{*}\leq P_{\text{max}}\;R^{2}-P_{\text{min}}R \end{array}$$

and the second inequality in (16) holds because

$$\xi_{2}\in \left(0,\frac{1}{R\left(P_{\text{max}}\;R-P_{\text{min}}\right)}\right)$$

and the third inequality in (16) holds because

$$\xi_{1}\in \left(0,\frac{1}{R^{2}-R+1}\right]$$

Inequality (16) contradicts the fact that (S^*^, P^*^, Γ^*^, W^*^, E^*^) is an optimal solution to the single-stage problem. Therefore, we should have S^*^ ≤ *S*^+^. On the other hand, (S^*^, P^*^, Γ^*^) satisfies constraints (6b)–(6f) since (S^*^, P^*^, Γ^*^, W^*^, E^*^) is an optimal solution to the single-stage problem. This means that (S^*^, P^*^, Γ^*^) is also a feasible solution to problem (6a)–(6f). Since (*S*^+^, **P**^+^, **Γ**^+^) is an optimal solution to problem (6a)–(6f), we have S^*^ ≥ *S*^+^. Together with the fact that S^*^ ≤ *S*^+^, it can be concluded that S^*^ = *S*^+^.

In the second step, we prove that the solution to single-stage problem (11a) and (11b) and the solution to problem (9a)–(9c), the second stage of the three-stage problem, will give the same negative utilization. We again use proof by contradiction. Suppose (**P**^#^, **Γ**^#^, *W*^#^) is an optimal solution to problem (9a)–(9c) associated with the minimum frame size S^*^, and W^*^ > *W*^#^. Since both W^*^ and *W*^#^ are integers, we have

$$W^{*}-W^{\#}\geq 1$$

Define

$$E^{\#}=\sum_{k=1}^{R}\sum_{m=1}^{C}\sum_{n=1}^{S^{*}}{\left(P_{k}^{m,n}\right)}^{\#}$$

Since (S^*^, **P**^#^, **Γ**^#^, *W*^#^, *E*^#^) satisfies (9b) and (9c), it also satisfies (11b). Thus, it is a feasible solution to the single-stage problem with the objective value being

(17) $$v^{*}=S^{*}+\xi_{1}\left(W^{*}+\xi_{2}E^{*}\right)>S^{*}+\xi_{1}\left(W^{\#}+\xi_{2}E^{\#}\right)=v^{\#}$$

The inequality in (17) holds because

$$\begin{array}{r} W^{*}-W^{\#}\geq 1\\ \xi_{2}(E^{\#}-E^{*})<1\\ \xi_{1}>0 \end{array}$$

Inequality (17) contradicts the fact that (S^*^, P^*^, Γ^*^, W^*^, E^*^) is an optimal solution to the single-stage problem. Therefore, we should have W^*^ ≤ *W*^#^. On the other hand, (S^*^, P^*^, Γ^*^, W^*^) satisfies constraints (9b) and (9c). This means that (S^*^, P^*^, Γ^*^, W^*^) is also a feasible solution to problem (9a)–(9c). Since (S^*^, P^*^, Γ^*^, W^*^) is an optimal solution to problem (9a)–(9c), we have W^*^ ≥ *W*^#^. Therefore, it can be concluded that W^*^ = *W*^#^.

In the third step, we prove that the solution of single-stage problem (11a)–(11b) and the solution to problem (10a)–(10d), the third stage of the three-stage problem, will give the same normalized energy consumption. We still use proof by contradiction. Suppose (**P**^†^, **Γ**^†^, *E*^†^) is an optimal solution to problem (10a)–(10d) associated with the minimum frame size S^*^ and the negative utilization W^*^, and E^*^ > *E*^†^. Since (S^*^, **P**^†^, **Γ**^†^, W^*^, *E*^†^) satisfies (10b)–(10d), it also satisfies (11b). Thus, it is a feasible solution to the single-stage problem with the objective value being

(18) $$v^{*}=S^{*}+\xi_{1}(W^{*}+\xi_{2}E^{*})>S^{*}+\xi_{1}(W^{*}+\xi_{2}E^{†})=v^{†}$$

The inequality in (18) holds because

$$\begin{array}{r} \xi_{2}(E^{*}-E^{†})>0\\ \xi_{1}>0 \end{array}$$

Inequality (18) contradicts the fact that (S^*^, P^*^, Γ^*^, W^*^, E^*^) is an optimal solution to the single-stage problem. Therefore, we should have E^*^ ≤ *E*^†^. On the other hand, (S^*^, P^*^, Γ^*^, W^*^, E^*^) satisfies the constraints (10b)–(10d). This means that (S^*^, P^*^, Γ^*^, W^*^, E^*^) is also a feasible solution to problem (10b)–(10d). Since (S^*^, **P**^†^, **Γ**^†^, W^*^, *E*^†^) is an optimal solution to the problem (10b)–(10d), we have E^*^ ≥ *E*^†^. Therefore, it can be concluded that E^*^ = *E*^†^.

From the preceding three steps, it can be concluded that three-stage optimization problem (6a)–(6f), (9a)–(9c) and (10a)–(10d) is equivalent to single-stage problem (11a) and (11b).

Both the three-stage and the single-stage optimization problems are always feasible if the desired interrogation range is acceptable because each reader can be separately scheduled at a time slot even in the worst case. At this time, the frame size and the utilization is identical with the number of readers. For the feasibility, the maximum output power should be large enough to achieve the desired interrogation range. In the next section, we present the numerical results from the software package LINGO [30], which solves the MILP problems.

## 5. Numerical Results

**Figure 2.**
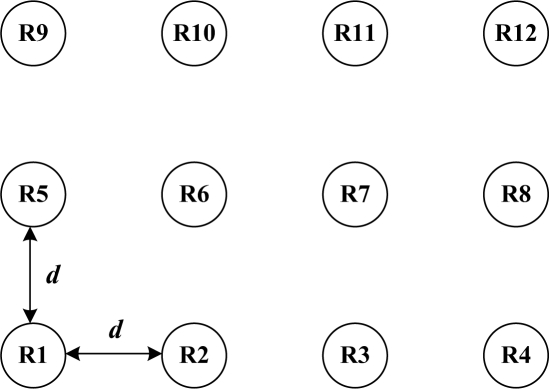
RFID reader deployment.

Consider a passive RFID system as shown in Figure 2. In the RFID system, each reader has an omnidirectional antenna and shares four channels. The distance between adjacent readers is *d* meters. The desired interrogation range for the readers is set to 1 meter. The maximum output power of each reader, *P*_max_, is set to 1 watt [21]. For the wireless signal propagation model, we assume that there is no shadowing or fading, and the signal power at the receiver is attenuated due to path loss with the path attenuation exponent being equal to 2. For the spectrum mask level, the transmit mask of a dense-reader environment in CEPT [11] is used. All the parameters used in the evaluation are shown in Table 1. The software package LINGO is used to find the solutions to the three-stage problem and the single-stage problem.

Tables 2 and 3 show the optimal solutions when *d* is set to 5 m and 15 m, respectively. In the system where *d* is set to 5 m, each reader has to wait at least four time slots for the next service. The major reason for the service interval is adjacent channel interference. As shown in Table 2, there are no pair of readers which can use adjacent channels except that they are diagonally placed at the corners, *i.e.*, readers 1 and 12, readers 4 and 9. Thus, at most three readers can be active at the same since four channels are assumed in the system. On the other hand, when *d* is set to 15 m, each reader needs to wait at most two time slots only for the next service. This is because when the distance between a pair of readers is sufficiently large, they may not experience the adjacent channel interference.

According to theorem 1, the three-stage problem should produce the same optimal solution as that of the single-stage problem. To show that how the three-stage optimization sequentially optimizes the system, we show the results obtained in each stage of the three-stage optimization and compare them with the single-stage solutions. To clearly compare them, we inactivate some readers and find the solutions.

Tables 4–6 respectively show the solutions to the first, second, and third stages of the three-stage problem in a case with inactive readers R7 and R9 where *d* is set to 15 m. The minimum frame size is derived in the first stage. Although ten readers, instead of twelve, are competing with each other, each reader still has to wait two time slots for the next service. This is because at most four readers can be scheduled in a time slot due to large co-channel interference. According to [3], each reader with the output power of 1 watt and the interrogation range of 4.25 m should be spatially separated at least 1,200 meters to prevent the co-channel interference effect. Since ten readers are deployed relatively close to each other in the system, the frame size for the system cannot be less than three. The reader utilization is maximized in the second stage while maintaining the frame size obtained from the first stage. Since four readers can be scheduled in a slot and a frame consists of three slots, the maximum reader utilization is twelve. In the third stage, the total signal power consumption of the readers is minimized while maintaining the optimality found in the previous stages. We observed that the single-stage problem and the three-stage problem produced the same results.

Since the proposed optimization problems should be solved in a centralized manner, the processing time is important for practical use. We simulated the software package LINGO on a PC with 2.13 GHz CPU, 2 GB RAM, Windows XP to solve the problem. It took The time needed by the PC to derive the solution to the single-stage problem and the solution to each stage in the three-stage problem was approximately three seconds and one second, respectively. Therefore, the proposed design can be effectively applied to small- or medium-size RFID systems.

## 6. Comparative View of the Anti-Collision Approaches

Most anti-collision approaches prevent reader-to-tag and reader-to-reader collisions by distributing operation time among the readers or dynamically assigning frequencies to the readers. The early approaches such as Colorwave, HiQ and LBT cannot fully utilize the potential capacity of RFID systems due to the heuristic nature of these approaches. Though there are several studies, such as Enhanced colorwave, Slotted-LBT, RS-LBT, DFSA, to remedy the shortcomings of the early works and multi-channel approaches using extra control channel, their performance is not satisfactory yet [14,23]. This motivates the emergence of optimization-based approaches such as FDFA/SDFA, DIA, DAPC/PPC and RA-GA [12,23,25,28,29].

In general, the optimization-based approaches develop elaborate models for the reader-to-tag and reader-to-reader collision problems and achieve their respective goals while minimizing collisions. FDFA/SDFA and DIA aim to achieve max-min fair channel allocation among the readers and dynamically allocate communication channels to the readers. DAPC/PPC and RA-GA aim to maximize the overall coverage area of the system while maintaining a desired read rate. To achieve the objective, DAPC/PPC controls the output power of readers and RA-GA uses a combination of FDMA and TDMA. Although such approaches formulate the optimization problems to achieve the goals, they have difficulty in finding optimal solution due to non-convex optimization problem [12], high complexity [25,28,29]. Thus, they approximate to the optimum using iterative update method [12,28,29] or heuristic search [25]. On the other hand, the proposed optimization approach is formulated as an MILP and the optimal scheduling solution can be simply derived with an LP solver [30]. Furthermore, most of the other approaches achieve a single objective with a restricted arbitration metric and they do not fully prevent the collision problem of RFID systems [12,23]. In contrast, the proposed approach achieves the three objectives by adapting various arbitration metrics such as time, channel, power while maintaining the required interrogation ranges of readers without any collision. Depending on the applications, it is also possible to use one or two metrics to optimize the system, *i.e.*, fixed power or single channel environment.

As a centralized approach, the proposed optimization design requires information on distances between the readers and computation time to derive the solution. For the formulation of the optimization model, we assume that there is no shadowing or fading, and the signal power at the receiver is attenuated due to path loss with the path attenuation exponent being equal to two. Furthermore, the computation to find solution may not be consistent with mobile RFID networks. Therefore, the solution from the proposed approach is appropriate for stable RFID networks with stationary readers, rather than mobile readers.

## 7. Conclusions

In this paper, we surveyed and classified a series of countermeasures to reader collision problem. Then, a novel MILP based cross-layer optimization problem for RFID reader arbitration was proposed, and its interaction with resource scheduling and power control was also derived. To formulate the problem, the reader-to-reader interference model was renovated with consideration of both resource scheduling and power control. Based on the priority of the multiple objectives, to sequentially optimize the system, our cross-layer design basically consists of three stages. Since it is cumbersome to derive the final solution due to the nature of the multi-stage problem, we presented an equivalent single-stage problem and proved the equivalence between the three-stage problem and the single-stage problem. Through the numerical results, we showed the effectiveness of our approach.

The main contribution of this paper is threefold: (i) For a UHF RFID system, we mathematically modeled the system requirements as linear equations and designed the MILP based optimization problem, (ii) we provided insights into how to arbitrate RFID readers with consideration of resource scheduling and power control, and (iii) we explained how to make the three-stage problem into an equivalent single-stage problem with more compact and concise mathematical form by properly assigning a weight to each objective. The proposed design can be easily extended to the analysis of various interferences from RFID readers or other systems such as short-range devices.

## Figures and Tables

**Table 1.** Evaluation Parameters.

Parameters		Values
Operating Frequency		915 Mhz
Channel bandwidth		500 kHz
Target SINR (BER ≤ 10^−5^)		11.6 dB
Tag threshold level (*P*_TH_)		−15 dBm
Tag’s power reflection coefficient (*E*_tag_)		0.1
Fading coefficient (*h*)		1
Normalized spectrum power (α_BW_) - FM0 code		0.86
Background noise (*N*_0_)		−60 dBm
Antenna Gain (*G*_T_ = *G*_R_)		6 dBi
Spectrum mask - CEPT dense-reader env.	*β*(0)	0 dBc
	*β* (1)	−30 dBc
	*β* (2)	−60 dBc
	*β* (≥ 3)	−65 dBc

**Table 2.** The optimal solutions when d = 5 m.

Frame size	Reader utilization	Energy consumption	Time slot	Scheduled reader (channel number, power(mW))
5	12	0.572W	1st	R1(1, 97), R6(4, 23), R12(2, 97)
			2nd	R4(4, 97), R9(3, 97), R7(1, 23)
			3rd	R2(1, 23), R8(4, 23)
			4th	R5(4,23), R11(1,23)
			5th	R3(4, 23), R10(1, 23)

**Table 3.** The optimal solutions when d = 15 m.

Frame size	Reader utilization	Energy consumption	Time slot	Scheduled reader (channel number, power(mW))
3	12	0.408 W	1st	R1(2, 36), R3(4, 36), R10(3, 39), R11(1, 29)
			2nd	R2(4, 33), R4(3, 35), R7(1, 36), R9(2, 34)
			3rd	R5(3, 31), R6(1, 34), R8 (4, 31), R12(2, 34)

**Table 4.** The optimal solutions to the first stage (6a)–(6f) where readers R7 and R9 are inactivated and d = 15 m.

Frame size	Reader utilization	Energy consumption	Time slot	Scheduled reader (channel number, power(mW))
3	10	3.234W	1st	R1(1, 24), R4(3, 1000), R6(4, 1000)
			2nd	R2(4, 32), R8(3, 40), R10(2, 42), R12(1, 35)
			3rd	R3(3, 31), R5(4, 30), R11 (1, 1000)

**Table 5.** The optimal solutions to the second stage (9a)–(9c) where readers R7 and R9 are inactivated and d = 15 m.

Frame size	Reader utilization	Energy consumption	Time slot	Scheduled reader (channel number, power(mW))
3	12	1.825 W	1st	R4(3, 36), R5(2, 46), R8(1, 31), R11(4, 32)
			2nd	R1(2, 238), R4(1, 999), R6(4, 37), R12(3,62)
			3rd	R2(4, 128), R3(1, 128), R8(2, 76), R10(3, 75)

**Table 6.** The optimal solutions to the third stage (10a)–(10d) where readers R7 and R9 are inactivated and d = 15 m.

Frame size	Reader utilization	Energy consumption	Time slot	Scheduled reader (channel number, power(mW))
3	12	0.386 W	1st	R1(3, 30), R4(4, 27), R6(1, 31), R12(2, 33)
			2nd	R5(3, 31), R8(2, 34), R10(1, 31), R12(4, 27)
			3rd	R2(4, 29), R3(2, 41), R11(1, 35), R12(3, 37)
